# Careers in ecstasy use: do ecstasy users cease of their own accord? Implications for intervention development

**DOI:** 10.1186/1471-2458-8-376

**Published:** 2008-10-28

**Authors:** Gjalt-Jorn Ygram Peters, Gerjo Kok, Herman P Schaalma

**Affiliations:** 1 Department of Work and Social Psychology, Faculty of Psychology and Neuroscience, Maastricht University, Maastricht, The Netherlands

## Abstract

**Background:**

Ecstasy (MDMA, 3, 4-methylenodioxymethamphetamine) use is widespread in the Netherlands, with a lifetime prevalence of 4.3%, and two-thirds of dance party visitors being ecstasy users. However, research into Dutch ecstasy use patterns is lacking. In addition, recent studies suggest that ecstasy users cease their use automatically, which implies that interventions would do better to better focus on the promotion of harm reduction strategies than on inducing cessation. The current study addresses this process of ecstasy cessation.

**Methods:**

32 participants from the Dutch dance scene were interviewed, and the results were systematically analysed using NVivo.

**Results:**

Most ecstasy users had started to use out of curiosity. During use, users applied a host of harm reduction strategies, albeit inconsistently and sometimes incorrectly. Most users appeared to cease ecstasy use automatically because of loss of interest or changing life circumstances (e.g. a new job or relationship).

**Conclusion:**

It appears that cessation of ecstasy use is largely determined by environmental variables and not by health concerns. This supports the idea that health promotion resources are better spent in trying to promote consistent and correct application of harm reduction practices than in trying to induce cessation.

## Background

Ecstasy (MDMA, 3, 4-methylenodioxymethamphetamine) use is potentially damaging to one's health [1-3]. A number of recent reviews and meta-analyses have summarized the growing body of research, finding ecstasy use to be associated to poorer neurocognitive functioning in a number of domains [1-5]. In addition, many users report negative effects that they attribute to their ecstasy use, such as decreased concentration, depression, insomnia and fatigue [6]. Nonetheless, ecstasy use remains prevalent. In the Dutch general population in 2005, 4.3% of all men and women between 15 and 64 had ever used ecstasy, 1.2% had used the past year, and 0.4% had used the past month [7]. Although these prevalence rates are lower than those of cannabis (22.6% lifetime, 5.4% last-year, and 3.3% past-month use), of dance event visitors, around two-thirds use ecstasy [8,9], whereas less than half use cannabis [8]. With over 13% of the 15–35 year-olds in the Netherlands visiting at least one dance event each year [10], this high prevalence in the dance scene is worrying.

Although it is generally acknowledged that prevention activities are needed to reduce the detrimental health effects of ecstasy use, to date there is a lack of formative research that would enable the theory- and evidence-based development of such interventions [11,12]. Recent research syntheses of ecstasy use correlates suggest that ecstasy cessation may be rather difficult to accomplish, since many ecstasy users may cease their use because of external events, such as a change in life circumstances (e.g. new job, relationship) or interference of use with 'normal life' [11,13]. This might imply that a harm reduction approach could be more effective in promoting the health of ecstasy users than interventions promoting ecstasy cessation. This suggestion is further supported by the lack of effects of attempts to promote ecstasy use abstinence [14].

A recent meta-analysis [11] of the determinants of ecstasy use and related behaviour concluded that as yet, there has been no quantitative research into the determinants of ceasing ecstasy use, and that the determinants of *using *ecstasy do not seem to lend themselves well for health promotion interventions (quantitative research was defined as research analysing the significance and strength of associations between determinants and intention or behaviour). A subsequent qualitative review [13] showed that people's reasons to cease ecstasy use *have *been studied qualitatively (e.g. in studies using interviews or univariate analyses), and these reasons differ from reasons to use or to initiate use. An important reason to cease use was a change in life circumstances. Four of the ten studies reporting on reasons for cessation were conducted in Australia [15-18], two in the United States [19,20], one in the United Kingdom [21], one in Germany [22], and two using the internet, with respondents from multiple countries [23,24].

In Australia, the topic was touched upon in four qualitative studies. The first study was conducted by Solowij et al. [17], who reported the following reasons why one-to-three time users had not used again: it did not live up to their expectations, wariness regarding the effects, financial reasons, or having found the experience boring or unpleasant. Topp et al. [16] found that reasons for wanting to reduce use were financial difficulties, physical health effects, psychological problems, occupational problems, to improve their quality of life, relationship problems, and feeling dependent on ecstasy. Gourley [18] found that "interviewees attributed their declining use to the fact that they were going to raves and other social events less often and were therefore growing out of the ecstasy scene" (p. 62). Hansen et al. [15], stated that "significant changes in user patterns were generally linked to a change in life circumstances" (p. 190).

In the United States (US), Carlson et al. [20] reported a qualitative study and specifically detailed three cases. In one case, an interviewee had tried ecstasy twice: the first time, she found the experience unpleasant; the second time, she experienced nothing. Then, after college, she got a job and planned to get married, and commented, "Since I've been out of college, I've lost touch with it". Levy et al. [19] reported a variation of motivational factors related to quitting ecstasy use: negative personal experiences while using ecstasy, health concerns, addiction/tolerance, money, loss of interest, negative observations of others using ecstasy, and fear of legal consequences.

In Germany, Soellner [22] asked for motives to cease use, and found that 75% endorsed "fear of reduced efficiency", 62% endorsed "fear of damage to health", and 37% endorsed "addiction". Another 44% stated that they stopped using without special reasons. However, Soellner indicated that it remains unknown whether the reported fears were related to health promotion efforts or to one's own experience.

In the United Kingdom (UK), Verheyden et al. [21] asked ex-users to rate the importance of a list of possible reasons for cessation. The most highly rated reason was "MDMA quality" (6.3), followed by "long-term mental health" (5.8), "not enjoying drug" and "depressed" (both 5.6), "paranoia" (5.4), "anxiety" (5.3), "memory" and "stopped clubbing" (5.1).

Gamma et al. [24] asked respondents in a web-survey whether they would cease ecstasy use if it caused problems, and found that although 30% would definitely cease and 30% would maybe cease, 16% were not sure, 18% would perhaps continue, and 7% would definitely continue. This illustrates that even when users experience problems themselves, this does not guarantee cessation. Rodgers et al. [23] reported that the most frequently reported reason to cease ecstasy use was "moving on" (16%), followed by "negative effects" (14%).

None of these studies has taken place in the Netherlands, and it is questionable whether their results are valid for the Dutch situation. Because of the relatively liberal drug policy in the Netherlands, drug use patterns may differ from those in other countries, particularly regarding the application of drug use harm reduction strategies [25-28]. In addition, different dance drugs are used in the Netherlands than in other countries [29]. For example, methamphetamine use is prevalent in Australia [30] and the US [31], but virtually nonexistent in the Netherlands [32,33]. LSD use shows a similar but less pronounced pattern [8,34]. As recreational drugs have been shown to be each others substitutes [35], these differences may affect ecstasy use patterns. To date, there is hardly any qualitative information available about Dutch ecstasy users, with only one study reporting Dutch data. Although these results do point in the direction of automatic cessation of ecstasy use, they are based on an unpublished source [36].

In order to explore ecstasy use patterns in the Netherlands, and in particular, ecstasy cessation, we conducted a qualitative study among Dutch dance scene members. Ecstasy users, as well as non-users and ex-users, were recruited in order to enable balanced data collection regarding the three periods that seem to comprise an ecstasy use career: initiation, regular use and cessation. Although snow-balling techniques are frequently used in illicit drug research and there is some evidence that snow-balling [37] and respondent-driven sampling [38] may provide representative samples, we decided to recruit participants at dance events to ascertain recruitment of regular dance event visitors and to be able to control the inclusion of non-users, users and ex-users. In addition to ecstasy use careers, we addressed harm reduction strategies employed by ecstasy users. The current paper provides a summary of the results of these interviews.

## Methods

In April and May 2004, we recruited 160 participants among visitors of three dance events in the Netherlands. Visitors were approached in the chill out areas and asked whether they would be willing to participate in a qualitative study on ecstasy use in exchange for monetary compensation. Visitors who agreed to participate were asked to fill out a small form where they provided contact information and information on their use of ecstasy, speed, cocaine, GHB, ketamine, ephedra and poppers. Specifically, they indicated whether they used each drug and if so, how frequently. If they did not use a drug, they indicated whether they had used the drug in the past but no longer used it now (ex-use) or whether they had never used the drug (non-use).

Afterwards, the 160 potential participants were called randomly. Although a minority of phone numbers turned out to be invalid, the majority of the potential participants that were invited agreed to participate in the study. Therefore, it was not necessary to contact all potential participants. The most common reason to refuse participation was inconvenience of the interview locations and times. Of the 32 participants that were recruited, 24 participants filled five focus groups, and 8 people were invited for individual interviews. Two non-using participants scheduled for individual interviews turned up together, and the interviewer interviewed them simultaneously. The other individual interviews were conducted with a non-user, three users and two ex-users. Of the five focus groups, one consisted of a non-user, two users, and an ex-user; one of a non-user, three users, and an ex-user; two of three users and two ex-users; and one of two users and three ex-users. The choice to combine focus groups and individual interviews was based on the lack of evidence as to how the interview method would affect the discussions about ecstasy use in this population. Combining both methods allowed provisional exploration of potential differences between the two methods.

Because both urban and rural participants were recruited, the focus group discussions (FGDs) and interviews were conducted at three locations throughout the Netherlands. Two trained interviewers conducted the sessions, using both a detailed interview/FGD scheme and a shorter topic list. These documents determined in which order the topics would be discussed, starting with neutral issues like music, going out and use of alcohol, tobacco and cannabis. Subsequently, ecstasy use was discussed, particularly the initiation of use, fluctuations in use, applying harm reduction practices, and cessation. The topics covered in the interviews partly depended on whether the interviewee was a non-user, a user or an ex-user, or, in the case of FGDs, on the group composition. Permission to perform this investigation was granted by the Ethical Committee Psychology of Maastricht University (the ECP).

The FGDs and interviews were transcribed and imported into QSR NVivo1.3 [39]. This allowed for coding of fragments using a flexible set of categories. Although these categories were initially based on concepts from the Theory of Planned Behaviour [40], a theory that has been found useful in explaining ecstasy use [41,42], it turned out that categories were so quickly adjusted and refined as the coding process progressed, that these initial categories hardly guided the coding process. The relevant and recurring categories that emerged will guide the description of the results. When applicable, results will be illustrated with a quote from the participants, translated from Dutch.

## Results

Participants' average age was 21 years, with 38% being female, 22% identifying as religious, and 47% living in an urban area (defined as a municipality with over 100.000 inhabitants). Of the 32 participants, 5 had self-identified as non-users on the recruitment form, 16 as users, and 11 as ex-users. During the interviews, however, it became clear that not all self-identified ex-users had decided to never use ecstasy again. In fact, most 'ex-users' did not define ex-use as a decision to 'never use ecstasy again', but rather as the circumstance that they did not plan to use ecstasy in the foreseeable future. Few 'ex-users' decidedly ruled out any future use. Most ecstasy users used ecstasy once a month or less, and quite a few only used a few times a year. The only variable that seemed to be associated with user type was age, with non-users having a mean age of 18, while the mean age of both other groups was 22 (t = 3.00, p < .01). The individual interviews lasted on average 45 minutes, whereas the FGDs lasted on average two hours.

Two differences between individual interviews and FGDs were observed. First, participants of FGDs seemed to speak less freely than those interviewed individually; for example, non-users interviewed individually seemed more negative about drug use than those in focus groups. Second, compared to those interviewed individually, users in FGDs seemed to be more concerned about appearing in control of their drug use. However, these differences may have been a consequence of the heterogeneity of the FGDs (combining non-users, users and ex-users); more homogenous groups may not have exhibited these patterns.

Regarding 'regular ecstasy use', the ecstasy use-related behaviour that has been studied most thoroughly, no reasons were reported that have not yet been summarized in an earlier meta-analysis [11] and qualitative review [13]. Basically, ecstasy using participants confirmed the observation that people use ecstasy because it enhances the perceived experience of a dance party. Specifically, participants indicated that ecstasy provided euphoria, energy and connectedness with peers, decreased social inhibitions, and 'levelled' individuals with ecstasy-using friends. Participants revealed a strong norm against using ecstasy to cope with problems: several users indicated that people should only use ecstasy when feeling good. We will not elaborate on these and alike reasons for ecstasy use, in order to allow for a more in-depth consideration of career transitions (i.e. from non-use to use and from use to ex-use) and application of harm reduction practices.

### Not starting to use ecstasy

Non-using participants indicated five major reasons for refraining from ecstasy. First, they indicated fear for the direct and acute effects of ecstasy. Second, they reported the negative health effects as a barrier. Third, some participants indicated fear of addiction. Reported reasons for this fear included observing ecstasy-using friends who no longer visited parties without using ecstasy, perceiving oneself as particularly sensitive to addiction, and having a conviction that all drugs were destructive and addictive. The fourth reason to abstain from trying out ecstasy was related to such a conviction: for a number of non-users, their refraining from ecstasy did not seem to be the result of consideration of the consequences of ecstasy use, but rather of adherence to what seemed like a strict moral principle. The fifth reason was simply the belief that one did not need ecstasy to enjoy oneself, or more specifically, to 'act crazily', referring to ecstasy's disinhibiting effects. Non-users further revealed that they did not perceive any social pressure to engage in ecstasy use; most of them indicated that they had non-using friends. Moreover, they did not regard ecstasy use to be highly prevalent at dance parties.

### Initiation of ecstasy use

The interviews clearly identified curiosity about the effects as the main reason to start ecstasy use, often initiated by interaction with ecstasy-using friends. Interestingly, many users indicated that among users it is generally 'not done' to encourage non-using friends to try out ecstasy. Yet, at the same time, users seemed to exhibit an enthusiasm about the drugs' effects that, through peer modelling, seemed to have quite encouraged non-using friends. Although they were curious, most users indicated that they were also quite nervous about negative effects when they tried out ecstasy. One user explained how he wanted to try out ecstasy, but in the end, was too afraid of its effects (a second attempt did succeed):

"I have all my friends in that scene, so it happens automatically. The first time I had . . . I was going to do it, then I said: 'No, let's not after all', because I was afraid, for the effects and for a . . . Well, the second time I did do it, and, well, it was not disappointing, let's put it like that."

(21-year-old male ecstasy user, focus group discussion)

Another user indicated that she waited until she had a relationship with someone who she trusted sufficiently. It appeared that once non-users had become curious about ecstasy, they waited for or actively created the right circumstances for them to try it out. However, even then, most users still exhibited ambivalence in that they remained wary about potential negative effects (mainly unexpected acute effects):

"Yes, look, you know that it's bad for you, period! Smoking is bad, alcohol is bad, drugs are bad. Ehm, and everybody who uses drugs knows that, everybody is always a bit afraid, like, 'what I'm doing now, it's not completely ok', but it feels so good, so, and I live only once."

(25-year-old male ecstasy user, focus group discussion)

### During use: harm reduction strategies

Throughout their use, the large majority of ecstasy users remained quite aware of the health risks of ecstasy use, and most indicated that they applied a variety of harm reduction strategies. All users clearly seemed to be aware of the need to hydrate during use, and most indicated that they did so. Some participants, however, mentioned that they disliked drinking large amounts of water when they were high, and some others reported that they just tended to forget. A few users had the impression that alcoholic beverages and energy-drinks could also hydrate (whereas these actually dehydrate). Although most users indicated that they were aware of the need to eat during use, many revealed that they disliked eating while high, as ecstasy use causes a very dry mouth. Some users mentioned that they ate the fruit sold at dance events. To compensate for the low intake of food during ecstasy use, most users indicated that they tried to eat properly before use. However, it was generally believed that failure to do so would not have serious negative consequences. Accordingly, in situations of unplanned ecstasy use, many seemed quite willing to forego their intention to eat properly in advance. Moreover, a minority of users revealed that they took ecstasy on an empty stomach to increase its effects. Another harm reduction strategy that was generally acknowledged, was visiting the chill out with sufficient frequency to prevent a high body temperature. However, only a few users seemed to consider this important, and although most indicated that they visited the chill out occasionally, these visits did not seem guided by harm reduction motives.

All in all, our interviews suggest that most ecstasy users attempt to practice harm reduction strategies consistently, but that many were quite willing to let short-term pleasure prevail. Perhaps because of an awareness of their unreliable application of harm reduction strategies, many users claimed to consume healthy nutrients after use, such as nuts, milk and fruit, and in some cases vitamins. These nutrients were perceived as replenishing the body after drug use, and several users showed some lay conceptions of the serotonin depletion due to ecstasy use. Our interviews further revealed that many users tried to get enough sleep, both before and after use, in order to balance the health effects of ecstasy. Both the intake of specific nutrients and the assurance of enough sleep seemed expressions of a belief that ecstasy use damages one's body, and that rest and replenishment is needed to counterbalance the harmful effects of drug use.

Another important harm reduction strategy is verifying the pill contents, since the dose of MDMA (ecstasy's active component) varies greatly, and pills sold as 'ecstasy' may contain different substances [43]. Yet, pill testing did not seem to be a very popular harm reduction strategy. A few users indicated that they had used testing kits and some others mentioned that they had checked the internet for pill details (e.g., pillreports.com). However, the only reliable method to determine pill content is to have a pill tested in a laboratory. A Dutch government-funded organisation, the Trimbos Institute, organises over twenty pill testing facilities all-over the Netherlands. These are free or very cheap to use and anonymous. However, users indicates less use of these facilities than of testing kits and the internet. Major reasons for the unpopularity of pill testing were a trust in drug dealers or friends who provided ecstasy, as well as the perceived hassle to reach testing facilities.

Finally, many users indicated that they moderated their ecstasy use. Most users moderated the intensity of their use by planning how many pills they would like to take, and taking only that amount to parties. However, this strategy also seemed to be inspired by the possibility of being caught at the drug checks at dance events. Another frequently mentioned way to moderate the intensity of use was to start with a whole pill, but only take 'booster doses' of half a pill. Likewise, many users indicated that they moderated the frequency of their ecstasy use, and that they limited use to less than once a month or once every six weeks -one of the harm reduction guidelines promoted by health educators in The Netherlands. Most users had moderated their frequency of use from the start, but a minority reported that they had gone through a period of very frequent use, where their lives had revolved around dance events, before eventually diminishing their use.

### Ceasing ecstasy use

The ex-users that were interviewed reported three main reasons to cease ecstasy use: responsibilities, loss of interest, and experiencing acute serious effects. The most frequently reported reason to cease use was changing responsibilities. Ex-users indicated that they had reduced their drug use when they noticed that their use interfered with their job or study. Additionally, changing jobs, entering a relationship, or having children were mentioned as reasons to cease use. Interestingly, the interviews with users suggested that they anticipate ceasing ecstasy use, and many referred to their current use as a 'phase' in their lives:

"Look, in this phase of my life I'm just at school, I study, in this phase I don't see myself stopping [with use] . . . After that there's a new phase . . . And see, whether that's the reason . . . Or, or . . . Wife, children, job, I don't know . . . That depends, but . . . I think that in any case, I will have stopped before I get to 30 . . . That is kind of like a deadline . . .

(22-year-old male ecstasy user, focus group discussion)

Specifically, the age of thirty was repeatedly mentioned as some kind of 'hard deadline' for ceasing ecstasy use and visiting dance events. However, the fact that the ex-users in our study had been recruited at dance parties indicates that at least some ex-users continue visiting dance events even after having ceased ecstasy use.

The second reason to cease ecstasy use that was frequently mentioned was loss of interest. Ex-users indicated that they had reached a point where 'they had seen it all', implying that the novelty of ecstasy use and dance events fades, perhaps in interaction with the introduction of new responsibilities. Third, a few ex-users indicated that they had reconsidered their ecstasy use because of experiences with acute and severe negative effects, such as being hospitalised themselves or having experienced a hospitalised friend.

Our interviews further suggested that ceasing ecstasy use was not perceived as a difficult thing to do. Most users indicated that they were rather confident regarding their ability to cease their drug use. Some ex-users reported that once they had decided to cease, this had indeed had been easy. Some others, however, reported that cessation was hampered by their desire to be 'on the same level' as their ecstasy using friends:

"No, I don't see that [stop using while friends keep on using] happening . . . I have considered that numerous times. [...] At some point you're just standing there, and you're completely sober, and you think like: 'ok, that guy is completely off it,' and then you don't really enjoy it any more, then you might as well go home."

(22-year-old male ecstasy user, individual interview)

Also, dance events were generally experienced as more pleasant when ecstasy was used, and accordingly, some users reported that ceasing drug use would probably go together with ceasing to attend dance events.

### Not ceasing ecstasy use

The most worrying aspect of ecstasy use, potential long-term health damage, was not reported as a major reason for cessation. Although the interviews indicated that users were quite aware of the long-term health risks of ecstasy use, almost none of the users seemed to be worried about the long-term effects of their drug use. This lack of worry about long-term effects seemed to be related to the control users perceive to have over acute negative health effects, and the fact that almost nobody had experienced acute negative effects of their drug use -possibly due to the harm reduction strategies that were frequently employed- let alone long-term negative effects. Moreover, those who mentioned that they had experienced mild acute negative effects (e.g. a bad trip) tended to attribute such incidents to accidental factors like a bad pill. Participants who had experienced severe health problems among acquaintances or their friends, tended to attribute the problems to ignorance and misuse of ecstasy:

"What if a good friend of yours would end up in a hospital?"

"Then, eh, then I would first just ask how much he used. Because it's like this: for example, if I think he used too much, then it's just his own fault."

(21-year old male ecstasy user, focus group discussion)

## Discussion

First of all, our study suggests that ecstasy use among young people in the Netherlands has a strong social character. Many of the drug-using young people that we have interviewed, revealed that they had started using ecstasy because of curiosity and because they felt tempted by, and attracted to, the positive effects ecstasy seemed to have on their friends during dance events. They wanted to be part of the thrill their friends seemed to experience through ecstasy use; they wanted to be on the same level. And once they had experienced the thrill, it became common practice to continue ecstasy use while visiting dance events with friends. Generally, this meant using a few times a year, and rarely more frequently than once a month. For dance party visitors who refrained from ecstasy, the thrill of ecstasy use seemed not to outweigh their fear of ecstasy's direct effects and negative health effects. Moreover, non-users indicated that they did not need ecstasy to "freak out" at dance events. And, perhaps most importantly, they seemed to visit dance events with friends who also do not use ecstasy.

A second major result of our study is that ecstasy users seem to be well aware of the health consequences of ecstasy use, and of the harm reduction strategies they can employ to reduce or balance these consequences. Moreover, our study indicates that, although many users mentioned inconsequent and inferior harm reduction strategies, almost none of them had ever experienced acute health problems due to ecstasy use.

A third important outcome of our study is that ceasing ecstasy use seems to be something that 'just happens' due to circumstances, for instance because visiting dance events on ecstasy does no longer fit with other obligations, such as a job or study, or with changing social relationships. As such, ecstasy cessation hardly ever seems to be the result of a careful consideration of pros and cons, nor do negative health experiences seem to play a substantial role.

A fourth result of our study is that ecstasy users generally do not expect cessation to be hard, which is in line with the experiences of those who had actually ceased use. The only factor that seemed to hamper cessation to some extent seems the desire to continue visiting dance events with ecstasy-using friends.

These findings substantiate those reported by Ter Bogt et al. [36]: most ecstasy users seem to cease use of their own accord, either because it starts to interfere with their 'normal life', or because they lose interest. The findings also seem consistent with the results from other countries (i.e., Australia, the US, the UK and Germany), although a number of reasons have not been reported by the Dutch sample. Financial reasons, relationship or social problems, feeling dependent, and fear of legal consequences were not reported by the Dutch sample. The omission of financial reasons is likely a consequence of the low prices of ecstasy in the Netherlands, with pills being as cheap as 1 or 2 euro when bought in bulk [33]. Fear of legal consequences likely did not play a role because of the aforementioned liberal drug policy in the Netherlands (although this reason is not very prevalent in other countries, either). Finally, the low frequencies of ecstasy use may have prevented social and relationship problems, as well as feelings of dependence, from manifesting themselves. Paradoxically, these low frequencies of use may be an indirect consequence of the liberal drug policy as well [25-28]. This policy allowed harm reduction initiatives to flourish, and many users seemed to limit their use frequency conform the guidelines provided by Dutch harm reduction initiatives (e.g., Unity, a peer education project, and "uitgaan en drugs", an initiative from the Trimbos Institute).

These findings also corroborate earlier findings that ecstasy users' perceptions of ecstasy's risks approximate "its scientifically recognized risk relative to other drugs" [[24], p. 190], and that while acute negative effects may impact drug use, this should not be expected of the possibility of long-term neuro-toxic damage [24]. Non-users are aware of ecstasy's dangers, and it seems that among those that start using, this awareness does not disappear. This suggests that health educators have been successful in their endeavours to convey the health risks of ecstasy use, but that this information neither deters curious non-users, nor causes cessation in users.

The factors that *do *cause cessation, interference with 'real life' and loss of interest, seem hard to influence through health promotion interventions. What does this mean for future health promotion programs targeting ecstasy use? The link between ecstasy use and dance events suggests that banning dance parties might be an effective strategy to reduce ecstasy use. However, as the dance scene became popular through illegally organised dance events, this does not seem a viable solution. At illegal events, organised harm reduction initiatives would be precluded. Furthermore, ecstasy use may also shift to another context. In all, banning dance events would seem a harmful course of action. Second, increasing fear of ecstasy's health effects might help to discourage ecstasy use, but most users' estimates of ecstasy's dangers actually reflect the scientifically recognized risk. In addition, people have generally not experienced any risks themselves. Presenting risk information that is not supported by the scientific literature likely discredits the messenger. Moreover, fear-based information has been shown to be an ineffective intervention strategy [44].

Third, regarding ecstasy use prevention, intervening on non-users' fear of addiction may be successful. It may be possible to use the phenomenon that for ecstasy users, ceasing ecstasy use seems linked to ceasing attendance of dance events. This form of dependence may well be an undesirable outcome for non-users who consider trying ecstasy out, since in all likelihood, they will not want to find themselves in the situation where they have to give up attending dance events if they want to stop using ecstasy. This new argument to abstain may take away some of the casualness seemingly associated with trying ecstasy out. This argument can be combined with non-users' belief that they do not need ecstasy to have a good time, by informing potential users that trying out ecstasy might make them need the drug in order to have a good time.

The two other reasons to not start using ecstasy do not appear feasible intervention targets. First, because no publication has reported on the successful creation of negative moral norms regarding a target behaviour, there is no known method to establish a moral objection to (hard)drug use. Second, the fact that the main reason to initiate ecstasy use is curiosity, implies that non-users' trigger to start using ecstasy is unlikely to be a change in their belief that they do not 'need' the disinhibiting effects of ecstasy.

Because most users seem to cease regardless of health promotion efforts, health educators may be able to achieve more health benefits by promoting harm reduction strategies (HRSs). Although many users already attempt to apply these, not all users manage to correctly and consistently do so. In addition, many users are willing to neglect application when this interferes with their goals of having a good time [consistent with the hedonistic nature of the dance scene; [36]]. In fact, some users knowingly neglect certain HRSs, such as eating properly beforehand, to maximise drug effects. This has also been found for chilling out [45-47]. Thus, successful health promotion about HRSs has clear health potential. However, a problem for health promotion about harm reduction is a lack of evidence on which to base guidelines. Although there are a number of studies into which harm reduction strategies users employ [e.g. [48,49]], there are no studies documenting the effects of these practices. Until this becomes clear, training users' skills to implement intentions to apply HRSs may be beneficial. In addition, the implementation of harm reduction strategies may be enabled or enhanced by improved party conditions, for example by providing on site testing facilities, food that is edible with a dry mouth, free water distribution, organised chilling breaks, and prompts to drink, eat, and chill out.

The current study is limited in several ways. First, the study has a cross-sectional design. It would be interesting to see which patterns emerge when a group of participants is followed over time. The Dutch NeXT study provides a good example of a design that would allow this [50,51]. Also, Von Sydow et al. [52] have conducted a quantitative longitudinal study, and although they found that indeed, over time, many users automatically ceased or reduced their use, they did not examine reasons or determinants of these transitions. The second limitation is typical of qualitative research, which relies strongly on participants' self-reported reasons and self-reported causal links between reasons and behaviour. Although this qualitative methodology is particularly suitable for exploratory research, the downside is that quantitative verification of the results is required, the latter methodology being less dependent upon participants' introspective skills and allows statistical inference and thereby generalisation. A third limitation is that, although the employment of both FGD's and individual interviews provided preliminary indications as to differences in the way participants discussed their (non-)drug use, this issue has not been explored more thoroughly. It would be interesting to explore this topic in more depth.

Regardless of these limitations, this study contributes to growing evidence that health communication about the negative effects of ecstasy use may not be able to generate behaviour change [53]. Because of this, and the high probability that ecstasy users cease their use regardless of the efforts of health promoters, it seems wise for health promoters to focus on harm reduction.

## Conclusion

The most important reasons to start using were curiosity about and desire for ecstasy's effects and a desire to be on the same level as friends. Those who refrained from using indicated that they did not need ecstasy to "flip out" at parties, and that for them, the fear of ecstasy's direct effects and negative health effects outweighed the thrill, and perhaps more importantly, that they generally visited dance events with friends who do not use ecstasy. Most users did not used frequently, and although all users applied harm reduction strategies, these were often inferior and applied inconsistently. Ecstasy use cessation seemed prompted by changing life circumstances (job/study responsibilities, relationships, losing interest in dance scene), rather by consideration of pros and cons of use, and accordingly, potential health effects do not seem to play a substantial role. Most users do not expect cessation to be hard, and most ex-users agree. The only apparent complication emerged for users who, while trying to cease, continued to visit dance events with ecstasy using friends. Furthermore, there is evidence that health educators have successfully conveyed ecstasy's health risks, as most users' risk perception seems to approximate ecstasy's scientifically recognized risk.

Differences with other countries in the saliency of reasons to cease seem may be explained by the Netherlands' liberal drug policy, which allowed harm reduction initiatives to flourish: many users seem to limit their use frequency according to the guidelines provided by Dutch harm reduction initiatives Unity and Uitgaan & Drugs, and these low use frequencies may have prevented the social, relationship, health, and dependence problems, that were cited by users in other countries, from manifesting. Ecstasy prevention interventions may focus on the fact that once somebody tries out ecstasy, chances are that they will use at most or all dance parties they attend, thus developing some form of dependence. This may take away some of the casualness associated with trying out ecstasy. In general, however, it seems that harm reduction interventions have the greatest potential for achieving health benefits. Improving party conditions, for example by providing free water and organising breaks to chill out, may prove very beneficial. Finally, there is an urgent need for more research into the effectiveness of the different harm reduction strategies.

## Competing interests

The authors declare that they have no competing interests.

## Authors' contributions

GJYP conducted the study and the analyses and drafted the manuscript. GK conceived of the study, contributed to the interpretation of the data and critically revised the manuscript. HPS contributed to the interpretation of the data and helped to draft the manuscript. All authors read and approved the final manuscript.

## Pre-publication history

The pre-publication history for this paper can be accessed here:


